# The role of final-state interaction in tensor polarization effects of the reaction $\gamma d \rightarrow p n \pi ^{0}$

**DOI:** 10.1038/s41598-023-34555-4

**Published:** 2023-05-09

**Authors:** Vyacheslav Gauzshtein, Alexander Fix, Bogdan Vasilishin, Eed Darwish, Matvey Kuzin, Michael Levchuk, Alexey Loginov, Dmitry Nikolenko, Igor Rachek, Yuriy Shestakov, Dmitry Toporkov, Arseniy Yurchenko, Sergey Zevakov, Zakaria Mahmoud

**Affiliations:** 1 Institute of High Current Electronics, Tomsk, 634055 Russia; 2 National Research Tomsk Polytechnical University, Tomsk, 634050 Russia; 3 Physics Department, Faculty of Science, Taibah University, 41411 Medina, Saudi Arabia; 4 Physics Department, Faculty of Science, Sohag University, Sohag, 82524 Egypt; 5 Stepanov Institute of Physics, National Academy of Sciences of Belarus, 220072 Minsk, Belarus; 6 Institute of Applied Physics, National Academy of Sciences of Belarus, 220072 Minsk, Belarus; 7 Tomsk State University of Control Systems and Radioelectronics, Tomsk, 634050 Russia; 8 Budker Institute of Nuclear Physics, Novosibirsk, 630090 Russia; 9 Novosibirsk State University, Novosibirsk, 630090 Russia; 10 Physics Department, Faculty of Science, King Khalid University, 62529 Abha, Saudi Arabia; 11 Physics Department, Faculty of Science, New Valley University, El-Kharga, 72511 Egypt

**Keywords:** Experimental nuclear physics, Theoretical nuclear physics

## Abstract

Tensor analyzing-power components $T_{20}$, $T_{21}$, and $T_{22}$ for the reaction $\gamma d\rightarrow np\pi ^0$ have been studied for the first time in the photon energy range from 280 to 500 MeV. The data are extracted from the experimental statistics accumulated at the VEPP-3 storage ring in 2002–2003. The measured asymmetries are compared with the results of statistical simulations performed with the $\gamma d\rightarrow np\pi ^0$ amplitude from a spectator model, taking into account corrections for the final-state interaction. The comparison demonstrates quite good agreement between the experimental results and the theory.

## Introduction

Photoproduction of $\pi$ mesons on nucleons and nuclei is one of the main sources of information about nucleon resonances. The special role of these processes in meson-nuclear physics is due to certain advantages of using photons as sensitive probes. First, the electromagnetic interaction is well understood within the framework of quantum electrodynamics. Second, photons can penetrate deep into nucleons and nuclei, thus making it possible to obtain more complete information about their internal structure. This property distinguishes photoproduction from, for example, scattering of pions, which experience intensive absorption in a nuclear environment due to strong coupling to inelastic channels.

In the region of the photon energies below 500 MeV, several theoretical models were developed to study pion photoproduction on a deuteron, where the impulse approximation is typically used. In this model, the deuteron is actually considered as a system of two nucleons on which the pion is produced like on free nucleons apart from kinematic and binding corrections. The reason for this is the weak binding of the deuteron and its relatively large size. The reaction amplitude is then expressed in terms of photoproduction on a single free nucleon, whereas the second nucleon acts as a spectator. The final-state interaction effects associated with rescattering of the final particles are described in terms of two-body, *NN* and $\pi N$, *t* matrices.

In most cases, models developed according to this scheme provide consistent description of unpolarized differential and total cross sections, but demonstrate larger deviation for polarization observables. In this regard, polarization characteristics are often used as a sensitive test of various model approaches. It is also well known that polarization measurements give more complete information about the dynamics of the process under study, compared to what can be achieved with an unpolarized cross section. For these reasons, despite the general technical difficulties in conducting experiments with a polarized beam and/or target, measurement of polarization observables are among the most important parts of many research programs aimed at studying photonuclear reactions.

From the theoretical point of view, the influence of the final-state interaction (FSI) has been discussed in detail in a fairly large number of publications^1–5^. It should be noted that, unlike, for example, deuteron photodisintegration $\gamma d\rightarrow np$, where two-body mechanisms are of crucial importance, the incoherent reactions $\gamma d\rightarrow NN\pi$ are dominated by the single-nucleon mechanism. Although the corrections due to final-state interaction are, apparently, most important to the spectator model, their contribution is usually at the level of a few percent of the total cross sections. In particular, as it has been shown in the works cited above, interaction between the final nucleons in the neutral channel $np\pi ^0$ leads to an approximately 15 $\%$ decrease of the total cross section in the $\Delta (1232)$ region, which is in general agreement with the experimental results^6^. Other mechanisms in which both nucleons are involved (like, e.g., meson-exchange currents) play a minor role, unless the leading mechanisms are suppressed.

Despite many theoretical analyses and quite high precision of the available experimental results for $\gamma d\rightarrow np\pi ^0$, so far there are no experimental data that could be used to study those FSI features which are directly related to the dynamical properties of the interaction between the final particles. The reason is that the noticeable FSI effects which can be distinguished by comparing theoretical predictions with experimental data are mainly an artifact generated by the plane-wave approximation. For example, due to FSI, a sizable decrease of the pion angular distribution $d\sigma /d\Omega _\pi$ in the extreme forward direction^1^ is simply a trivial consequence of the fact that the resulting cross section contains non-physical contribution from the coherent channel $d\pi ^0$ if the plane-wave approximation is used for the final *np* system. The latter appears due to nonorthogonality of the plane wave $e^{i\textbf{q}\textbf{r}}$ and the wave function $\phi (\textbf{r}\,)$ of the coupled *np* system (the deuteron). As demonstrated in^3^, after eliminating this ghost admixture, the remaining FSI effect turns out to be relatively small. In other words, the significant influence of FSI in the reaction $\gamma d\rightarrow np\pi ^0$ is basically just an unavoidable consequence of using the plane-wave impulse approximation, so that it does not provide any interesting information about the role of *np* rescattering in this process.

One of the ways to minimize the influence of such ghost FSI effects is to study polarization asymmetries. The latter are expressed in terms of the ratio of different hermitian combinations of spin amplitudes, so that these undesirable effects, entering the numerator and denominator with approximately equal weights, are to a large extent cancelled. In addition, it is obvious that FSI should play a primary role in the kinematic regions that are characterized by a large momentum transfer and where the mechanisms with the participation of both nucleons become important. However, the available experimental data mainly cover the region of small momentum transfer, where the FSI effects are quite insignificant (after eliminating the mentioned influence of non-orthogonality of the wave functions). The only exceptions are the data for $\gamma d\rightarrow \pi ^+nn$^7^, obtained near the threshold, and the data for the distribution $d^2\sigma /(d\Omega _{\pi }\,dE_{nn})$ in the same reaction^8^, demonstrating a clear maximum at $E_{nn}\rightarrow 0$ coming from the $^1S_0$ virtual *nn* state.

In this work, we present experimental results for three components $T_{20}$, $T_{21}$, and $T_{22}$ of the tensor analyzing power for the reaction $\gamma d \rightarrow p n \pi ^{0}$. The data are extracted from the experimental statistics accumulated on the VEPP-3 electron storage ring in 2002-2003. VEPP-3 is an accelerator-storage complex located at the Budker Institute of Nuclear Physics, Novosibirsk. It is designed to accumulate and accelerate electrons and positrons. Presently, VEPP-3 is mostly used in various nuclear physics experiments with internal gas targets and as injector for the VEPP-4 accelerator. It contains the internal target equipment whose key element is the Atomic Beam Source with superconducting sextuple magnets, providing a flux of polarized deuterium atoms with high degree of tensor polarization and a negligibly small vector polarization admixture.

The results of measurements are compared with the theoretical predictions provided by statistical simulation based on the spectator model, which also takes into account the contribution of *NN* and $\pi N$ interaction in the final-state.

The paper is organized as follows. In the next section, the method and the formalism used to obtain the components $T_{2M}$ are described. In section “Results”, the data obtained in the present experiment are compared with the results of statistical simulation. A brief discussion of the results and conclusion are given in the last two sections.

## Research method

The present experiment was performed with an internal target filled with gaseous deuterium, only for which a high degree of tensor polarization can be obtained. A relatively small thickness of the target was compensated by a high beam current inside the accelerator chamber. A jet of polarized deuterium atoms entered the internal target from an atomic beam source (ABS) installed in the median plane of VEPP-3. At the exit of ABS, the degree of deuteron polarization was close to 100$\%$. A detailed description of the atomic beam source can be found in Ref.^9^.

A general expression for the cross section of pion photoproduction on a tensor-polarized deuteron reads

1 $$\begin{aligned} d\sigma = d \sigma _{0}\left\{ 1 + \frac{1}{\sqrt{2}}\,P_{zz}\left[ d_{00}^{2}\left( \theta _{H}\right) T_{20} - d_{10}^{2}\left( \theta _{H}\right) \cos \left( \phi _{H}\right) T_{21} + d_{20}^{2}\left( \theta _{H}\right) \cos \left( 2 \phi _{H} \right) T_{22}\right] \right\} , \end{aligned}$$

where $d \sigma _{0}$ is the corresponding unpolarized cross section, $T_{20}$, $T_{21}$, $T_{22}$ are the components of tensor analyzing power, and $d_{M^\prime M}^{J}$ are the Wigner *d* matrices:

2 $$\begin{aligned} d_{00}^2(\theta )=\frac{3}{2}\,\cos ^2\theta -\frac{1}{2},\quad d_{10}^2(\theta )=-\sqrt{\frac{3}{8}}\,\sin 2\theta ,\quad d_{20}^2(\theta )=\sqrt{\frac{3}{8}}\,\sin ^2\theta . \end{aligned}$$

    The orientation of the target polarization axis with respect to the direction of the photon beam is specified by the polar and azimuthal angles $\theta _H$ and $\phi _H$. The target polarization is determined by the degree of tensor polarization $P_{zz}$

3 $$\begin{aligned} P_{zz} = 1 - 3 n^{0}, \end{aligned}$$

where $n^{0}$ is relative population of the deuteron state having zero spin projection on the target-polarization axis.

**Figure 1:**
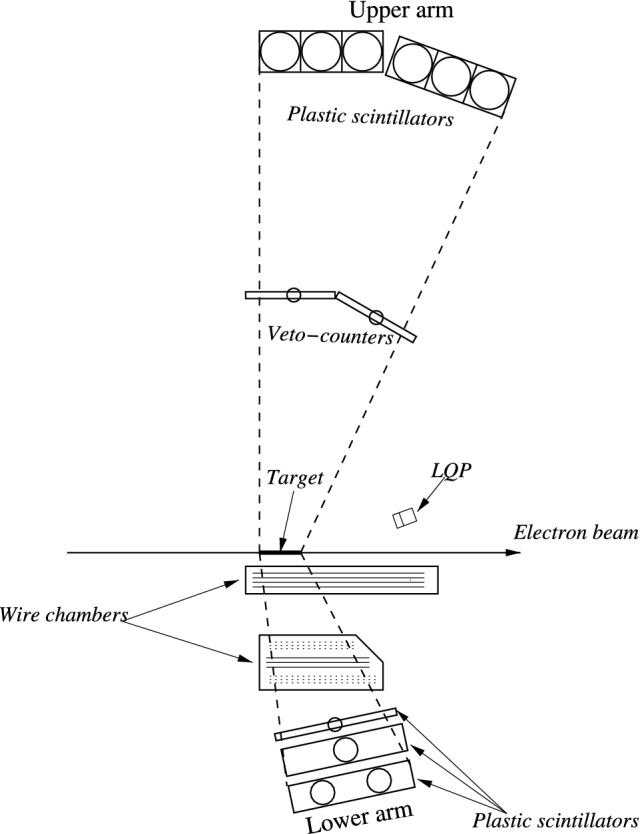
General scheme of the experiment.

The presented work reports on the analysis of data obtained from the experiment conducted in 2002–2003. The recoil proton and neutron were detected by coincidence of the two, lower and upper, systems of detectors (Fig. 1). The lower system, which was used to detect recoil protons, consisted of a set of drift chambers and three plastic scintillators. Recoil neutrons were detected in the upper system by using the time-of-flight method. Six thick scintillators were installed at a distance of 3 m from the target, and a thin scintillation counter was installed at a distance of 1.5 m. The polar angle for recoil protons and neutrons varied between $50^{\circ }$ and $90^{\circ }$, with their azimuthal angles being within $\pm 30^{\circ }$ for the lower arm and $\pm 12^{\circ }$ for the upper arm.

During the data collection, the polar angle $\theta _H$ was periodically changed to be one of the three values $54.7^{\circ }$, $125.3^{\circ }$ and $180^{\circ }$, whereas the azimuthal angle $\phi _H$ remained close to $0^{\circ }$ in all cases. The sign of the tensor polarization was switched every 30 s. Such a procedure allowed simultaneous measurement of three asymmetries with respect to the sign reversal of the tensor polarization:

4 $$\begin{aligned} a_i^{T}=\sqrt{2}\,\frac{N_i^{+}-N_i^{-}}{P_{zz}^{+}N_i^{-}-P_{zz}^{-}N_i^{+}}. \end{aligned}$$

    Here, $N_i^{+}(N_i^{-})$ is the number of events detected for *i*-th value ($i=1,2,3$) of the angle $\theta _H$ and the target polarization $P_{zz}^{+} (P_{zz}^{-})$. From Eqs. (1) and (4), the required expressions for all three components of the tensor analyzing power $T_{2M}$ ($M=0,1,2$) are obtained as

5 $$\begin{aligned} T_{20} = a^T_1,\quad T_{21} = \frac{\sqrt{3}}{4}\left( a^T_2-a^T_3\right) ,\quad T_{22} = \frac{\sqrt{3}}{2\sqrt{2}}\left( a^T_2+a^T_3\right) . \end{aligned}$$

    The details of the experimental setup, detectors for registering the reaction products, and the methodology adopted for identifying the reaction channel under study are given in Refs.^10–16^.

## Results

**Figure 2:**
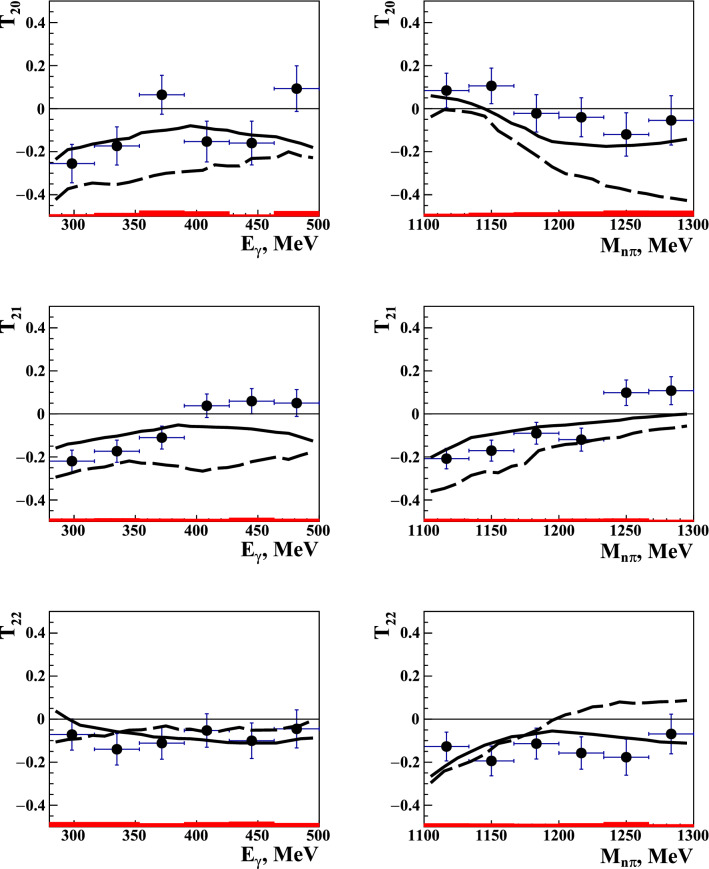
(Color online) The tensor analyzing-power components $T_{20}$, $T_{21}$, and $T_{22}$ for $\gamma d\rightarrow np\pi ^0$ as functions of the photon energy $E_\gamma$ (left panels) and the pion-neutron invariant mass $M_{\pi n}$ (right panels). The data points shown by filled circles represent experimental results of the present experiment, with their error bars are reflecting statistical uncertainties. The red bars underneath each data point reflect its systematic uncertainty. The solid (dashed) curves correspond to the results of simulation with (without) taking into account $\pi N$ and *NN* rescattering in the final-state.

The experimental results obtained for all three components $T_{2M}$, $M=0,1,2$, are presented in Fig. 2 as functions of the laboratory photon energy $E_\gamma$ and of the invariant mass $M_{\pi n}$ of the $\pi ^0n$ system. As seen, the asymmetries are quite small and do not exceed 0.2 in absolute value. Because the acceptance corrections for experimentally observed events $N^+$ and $N^-$ are canceled in the ratio (Eq. 4), they were neglected in the analysis of the experimental data, as well as for the simulated data. The magnitudes of statistical and systematic uncertainties for each data point can be seen in Fig. 2, illustrating a strong dominance of statistical uncertainties. The magnitude of the systematic uncertainties dominates by the uncertainty in determining the degree of the deutron tensor polarization.

The corresponding statistical-simulation results are plotted in the same figure. It was performed using the Monte-Carlo algorithm described in Refs.^17,18^, which makes it relatively easy to take into account complex boundaries of the experimental kinematic domain, as well as the inhomogeneity of the spatial distribution of the deuteron target.

Statistical simulation was carried out in full accordance with the experimental conditions, including the same constraints on the energies and emission angles of the final-state nucleons. To match the experimental target conditions, the components of the deuteron density matrix were simulated with the same probability, 1/6, for each of the six polarization states. Similar to the experimental events, the components $T_{2M}$ were extracted by using the same formulas (4) and (5) and identical intervals for averaging of the kinematic variables. Such an approach allows a direct comparison of the experimental and theoretical results.

**Figure 3:**
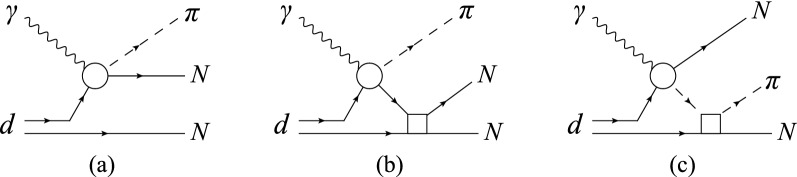
The pion photoproduction mechanisms considered in the present study: (**a**) quasi-free photoproduction, (**b**) *NN* interaction, (**c**) $\pi N$ rescattering.

To calculate the reaction amplitude that was embedded into the Monte-Carlo algorithm, we used the $\gamma d\rightarrow NN\pi$ model developed in Ref.^3^. The amplitude is built within the approximation in which the process on the deuteron is reduced to the sum of single-nucleon photoproduction amplitudes. As already mentioned above, for a process like $\gamma d\rightarrow NN\pi$, where the deuteron breaks up, such an approach is called as the spectator model (diagram (a) in Fig. 3). The elementary amplitude $\gamma N\rightarrow N\pi$ (shown as a circle in Fig. 3) was taken from the MAID2007 model^19^. It contains contributions from the nucleon born terms, *t*-channel vector-meson exchange, and a set of *s*-channel baryon resonances. The latter includes all resonances with masses up to 2 GeV that are classified with four stars in the Review of Particle Physics^20^.

Between the two mechanisms generating final-state interaction, the most important in the kinematical region of the present experiment is the nucleon-nucleon rescattering [diagram (b) in Fig. 3]. This is due to both a larger intensity of the *NN* interaction, compared to $\pi N$, and a fairly high kinetic energy of the final-state nucleons. An additional *NN* scattering effectively fills the missing-energy balance between the fast active nucleon and the spectator.

The $\pi N$ interaction is less significant here, primarily because the pion is unable to transfer any large amount of kinetic energy between the nucleons due to its small mass.

For the deuteron wave function, as well as for the *NN* scattering state, the separable version of the Paris potential from^21^ was adopted, in which the partial waves were taken into account up to $^{2S+1}L_J=^3\!G_3$. The calculation of the diagram with pion rescattering [diagram (c) in Fig. 3] was carried out using the separable model for the $\pi N$ amplitude from^22^ with all $\pi N$ partial waves up to $l=2$.

Note that in the present calculation, the final-state interaction effects were included only up to the first-order terms in the two-body *NN* and $\pi N$, *t* matrices, neglecting the higher order terms in the corresponding multiple scattering series. The latter could be taken into account, for example, within a three-body $\pi NN$ model, as it is done in^23^. As shown in^23^, despite the smallness of the contribution from higher-order multiple-scattering diagrams to the unpolarized cross section, it could still be important. The main reason for neglecting the higher-order terms is in a significant increase of the time required to run statistical simulations with three-body calculations. Thus the question about importance of the terms beyond the first order in the two-body *NN* and $\pi N$ rescatterings remains open.

This work also did not address the question about how much the results of simulation in Fig. 3 depend on a model used to construct the elementary amplitude $\gamma N\rightarrow N\pi$. In general, such dependence should not be very crucial because, in the energy range under the consideration, $E_\gamma < 500$ MeV, the existing multipole analyses for $\gamma N\rightarrow N\pi$ differ little from each other. However, because of quite specific kinematic conditions of the experiment (in particular, a large momentum transfer to the spectator nucleon), theoretical results may even be sensitive to small differences between the model amplitudes.

As shown in Fig. 2, taking in to account the final-state interaction effects significantly improves the agreement between the model predictions and the experimental data points, even if some features of the observed tensor asymmetries are not fully reproduced. This is especially important after taking into account quite high sensitivity of $T_{2M}$ to various model details, as well as to the special kinematic conditions of the present experiment that were discussed in the text above. Such an observation can be reviewed as an indirect confirmation of the general assumption that the spectator model, including *NN* and $\pi N$ rescatterings as the next order terms, may be considered as an adequate theory of the process under study.

## Conclusion

The present work reports on the first measurements of the tensor analyzing-power components $T_{2M}$, $M=0,1,2$, for incoherent $\pi ^0$ photoproduction on a deuteron in the range of the incident-photon energies from 280 to 500 MeV. The experimental results were obtained from analysis of the data accumulated on the VEPP-3 electron storage ring at the Budker Institute for Nuclear Physics in 2002–2003. The present results are compared to the predictions from statistical simulation performed with and without final-state rescattering, which demonstrates that taking such interactions into account significantly improves their agreement.

The results presented in Fig. 2 seems so far to be the only case when the importance of including interaction effects in the incoherent photoproduction of $\pi$ mesons on the deuteron is unambiguously and quantitatively established, which allows a conclusion that, in the given kinematical region, incoherent pion photoproduction is strongly influenced by FSI. Besides, the agreement observed between the present experimental results and model calculations indicates that a physical picture with quasi-free photoproduction on individual nucleons of a deuteron, accompanied by the final-state *NN* and $\pi N$ rescatterings, works well up to surprisingly high momenta transferred to the nuclear system.

## Data Availability

A request to receive the experimental and simulated data sets of the present work should be addressed to the corresponding author of the manuscript.
